# Pre and post diagnostic dementia care in four Scottish prisons

**DOI:** 10.1186/s40352-024-00294-5

**Published:** 2024-11-06

**Authors:** Rhoda MacRae, Natalie Chalmers, Debbie Tolson, James Taylor, Kirstin Anderson, Lindsay Thomson, Tom Russ

**Affiliations:** 1 Alzheimer Scotland Centre for Policy and Practice, School of Health and Life Sciences, University of the West of Scotland, Lanarkshire Campus, Glasgow, G72 0LH Scotland; 2 School of Health in Social Science, The University of Edinburgh, Teviot Place, Edinburgh, EH8 9AG Scotland; 3 School of Health and Life Sciences, University of the West of Scotland , Lanarkshire Campus, Edinburgh, G72 0LH Scotland; 4 Edinburgh Napier University, Sighthill Campus, Edinburgh, EH11 4BN Scotland; 5 Centre for Clinical Brain Sciences, Division of Psychiatry, University of Edinburgh, Edinburgh, EH8 9YL Scotland; 6 The State Hospitals Board for Scotland, Department of Forensic Psychiatry, Carstairs, UK

**Keywords:** Dementia, Older Prisoners, Care pathway

## Abstract

**Background and purpose:**

The number of older people in prisons is increasing across the globe. Many have poor physical and mental health, higher prevalence of head injury, cognitive impairment and dementia than found in community populations. Meeting the complex needs of this vulnerable group has become an increasing concern for prison and prison healthcare services. The aim of this multi method qualitative study was to investigate how men with diagnosed or suspected dementia were identified, assessed, and cared for in Scottish prisons. It also explored the lived experience of individuals being assessed for or diagnosed with dementia within four prisons. The data from twenty nine interviews was thematically analysed and used to collaboratively propose principles for dementia care in prison and present the resultant co-designed care pathway.

**Results:**

At the time of data collection almost all the men known to have a dementia diagnosis or suspected dementia had complex health and social care needs, and some were living with advanced dementia. Prison healthcare staff reported taking a ‘case by case’ approach to their pre- and post-diagnostic care. Meeting these prisoner’s needs was complicated by the absence of organisational leads for care of older adults or people with dementia and there was no pathway or model in place to guide staff. Prison healthcare teams often had difficulty accessing specialist community services to support diagnosis. There was a lack of dementia education and knowledge about how to provide pre and post diagnostic dementia care in this setting amongst staff. The findings arising from this research have informed the co-production of two important evidence informed innovations namely a Model of Care and a pre- and post-diagnostic Care Pathway.

**Conclusion:**

This research adds insights critical to understanding the adequacy of current approaches to meeting dementia related needs within the prison setting. To our knowledge this paper offers the first co-produced evidence informed pre- and post- diagnostic dementia care pathway and model of care for use in prisons. These could serve as tools for change that could enable prison healthcare staff to deliver the right care, at the right time, by the right people, and provide an opportunity to assess risk and plan care for the future.

## Background

Dementia is a clinical syndrome caused by illnesses such Alzheimer’s and vascular disease which manifests as progressive cognitive and functional impairment. Dementia also has a psychological, social and economic impact for people with dementia, their carers and families, and presents challenges for society and healthcare systems (SIGN 168, 2023). Illness progression is not fixed or predictable, and people can live for months and years with advanced dementia-specific palliative care needs (Reisberg., et al. 2006; Hanson et al., 2019).

Prison healthcare in the UK is based on the principle of equivalence, which means that people detained in prison should receive equivalent standards of care – and achieve equivalent health outcomes – as the general population (House of Commons Health & Social Care Committee, 2018; UNODC and WHO Regional Office for Europe, 2013). Equivalence does not mean that all prisoners should be treated the same, yet it has been found that the principle of ‘sameness’ is applied to all prisoners regardless of need (Williams, 2012). Although, guidance outlining the health care services that should be provided in prison is available (NICE, 2018; HM Inspectorate of Prison Scotland, n.d.), previous reports have found prison health care understaffed and under resourced (House of Commons Health & Social Care Committee, 2018; Ismail, 2020; Mental Welfare Commission, 2022). Reforms have brought different health structures and processes but, the challenge of addressing health inequalities and equivalency of provision remains (Royal College of Nursing, 2016; Mental Welfare Commission, 2022; Gilling McIntosh et al., 2023).

Longer prison sentences and historical sentencing has contributed to the number of older people in prison increasing across the globe (Turner et al., 2018). In the UK, the over 60 age group has trebled in the last two decades (Ministry of Justice, 2022), and 44% of those over 50 years have been sentenced for sexual offences, many historical (Prison Reform Trust, 2022). There is no commonly accepted definition of old age in prisons. Public Health England suggests 50 years appears to be a threshold in widespread use (2017), however HM Inspectorate of Prisons Scotland have used 60 years (HMIPS, 2017). These lower age thresholds are based on suggestions that the health-related needs of prisoners are advanced by around 10 years, relative to people in the general population (House of Commons Justice Committee, 2019–21). Although older prisoners are a heterogenous group, two decades of research has highlighted that many have poor physical and mental health, often with accompanying complex health and care needs (Fazel et al., 2001; Maschi et al., 2012; Hayes et al., 2012; Mental Welfare Commission, 2022; Gilling McIntosh et al., 2023). The prevalence of head injury amongst prisoners is high (McGinley et al., 2019), increasing the risk of cognitive impairment with the associated neurobehavioural effects. These trends indicate this group may have unique health related challenges and needs (MacAllister et al., 2008; Worthington et al., 2017).

Older prisoners with complex health and social care needs create a challenge for prisons (Peacock et al., 2018; Wangmo et al., 2015). Existing approaches have been criticised for being inconsistent, institutionally thoughtless, and neglectful of the physical, emotional and social care needs of older prisoners (Crawley, 2005; Prisons and Probation Ombudsman, 2016; Williams, 2012; HM Inspectorate of Prisons and Care Quality Commission, 2018). Failings in the identification or management of deterioration in long term conditions has led to preventable deaths (Shaw et al., 2020). Many of the factors that compound the negative effects of ageing and dementia are exacerbated in prison, these are magnified further by the tensions and challenges of delivering effective healthcare while balancing concerns about risk and security (Prison and Probation Ombudsman, 2016). Older prisoners with cognitive impairment and physical health care needs, who are ‘old and quiet’ and or sentenced for sex offences are likely to be stigmatised in multiple ways, isolated and unsupported to live in dignity (HMIP, 2004), and as such are subject to symbolic annihilation (Fernandes et al., 2018). Very few prisons have implemented initiatives to support people with cognitive impairment. Some exceptions to this have been in the United States (Hodel and Sanchez, 2013) Australia (Baldwin and Leete, 2012) and England (Moll, 2013; Treacy et al., 2019), though the application of dementia-friendly principles for this highly vulnerable group were often not prioritised (Treacy et al., 2019).

Prevalence estimates of cognitive impairment and dementia in prisons vary. Estimates of cognitive impairment amongst prisoners over 50 years have ranged from 1.3% (Forsyth et al., 2020), 12% (Kingston et al., 2011), to 20% (Combalbert et al., 2018). In those over 55 years, Ahalt et al., found that 49% were cognitively impaired (with educationally adjusted lower cut off scores) (2018). Dementia prevalence amongst prisoners over 50 years also varies from 7.2% (Forsyth et al., 2020) to 20% in those over 55 years (Ahalt et al., 2018). Cognitive tests are not diagnostic on their own, and these studies may both under- and overestimate prevalence; validated screening tools for this population have yet to be developed (Ahalt et al., 2018; Forsyth et al., 2020). However, there seems to be widespread agreement that this population is likely to have higher prevalence of cognitive impairment and dementia than the general population.

Evidence suggests that dementia is an increasing concern for prison staff and prison healthcare practice and policy due an increasing older prison population and the complexity of their health and social care needs (Peacock et al., 2020; Brooke et al., 2020 and Du Toit et al., 2019). The consequences of not receiving dementia-related support may heighten fear and distress, deny access to appropriate healthcare and at worse facilitate a violation of human rights. Improved ways of recognising and managing prisoners with complex health needs including dementia is urgent (HMIPS, 2017; Gaston, 2018). Yet, there is little known about this population or how they are cared for in Scotland and internationally. A lack of evidence based recommendations risks ad hoc arrangements and perpetuates healthcare inequalities (Brooke et al., 2020). To ensure that the standards of care enshrined in Global Dementia Strategies and principles of equivalent healthcare are met for prisoners with dementia, evidence based guidance for this population is a priority.

## Methods

### Aim

The study aim was to identify and develop new effective ways to improve the health and well-being of the increasing numbers of older prisoners living with a diagnosed or suspected dementia. The objectives were to.

1. Describe the current referral, diagnostic and post-diagnostic healthcare pathways.
2. Understand how later life well-being is promoted and health risks reduced.
3. Construct case studies to exemplify the dementia lived and care experience.
4. Co-produce an evidence informed referral, diagnostic and post-diagnostic healthcare pathway.
5. Co-produce an evidence informed model of care to promote later life well-being and reduce health related risk.

### Design

This was a multi method qualitative study with three phases. Twenty semi-structured interviews with staff working in the four prisons in Scotland that housed the largest number of men over 65 years of age were conducted. Case studies were constructed from five semi-structured interviews with men with a diagnosed (*n* = 1) or suspected dementia (*n* = 4), four semi-structured interviews with individuals the men nominated and data from the men’s health care records (see Table 1 and data collection section for further details). Three stakeholder workshops using an adapted World Café Method™ (2024) to facilitate in-depth focussed conversations and harvest views about how pre- and post-diagnostic care for people with a diagnosed or suspected dementia could be operationalised and achieved were held.

**Table 1:** Case study characteristics

Case Number	Age	Referred by and reason	Diagnosed Dementia	Cognitive screen results	No of other health conditions	Nominated other
Case 1	63	Self ‘pre-sentencing cognitive issues’	No	Not documented	4	Prison officer
Case 2	68	Social work for ‘cognitive issues’	No	2022 MMSE 22/30 2022 MMSE 18/30	3	Prison officer
Case 3	67	Not documented	Yes	Cognitive screen 2020 score not documented	8	Mental Health Nurse
Case 4	79	Prison staff or ‘cognitive issues’	No	2021 ACE-R 68/100	6	Mental Health Nurse
Case 5	60	Prison staff for ‘communication, behaviour and memory issues’	No	2018 MMSE 22/30 2020 MMSE 25/30 2021 MMSE 22/30 2022 ACE-R 52/100 2022 ACE-R 51/100	6	Mental Health Nurse

^*^In both cognitive tests (MMSE, ACE-R); the higher the score denotes better cognitive functioning. An ACE-R score of 51/100 indicates significant cognitive impairment, an ACE-R score of 68/100 and an MMSE score of 18 indicates moderate cognitive impairment

### Access and recruitment

Scottish Prison Service (SPS) governors and senior teams arranged local access permissions and training to allow the research team to work safely in the prison environment. The NHS prison health board leads provided formal introductions to the Health Care Centre Managers of the four prisons. The Health Centre Managers provided the prison healthcare teams with participant information sheets via email or at staff meetings. Prison health care staff were invited to contact the research team independently of the manager or ask the manager to pass on their details to the research team. We asked the healthcare managers to identify men with a diagnosed or suspected dementia. We provided them with our inclusion and exclusion criteria, a participant information sheet, a ‘meet the researcher’ consent form and guidance to support them with explaining the study and asking the men for consent to meet the researcher. The inclusion criteria were that prison or healthcare staff deemed the person safe to be interviewed; had a functional command of English; capacity to provide informed consent, were willing to participate; had a dementia diagnosis or undergoing assessment for dementia; had more than one month of their sentence to serve.

### Sampling and sample

Purposive sampling was used to reach a range of prison health care staff from across the four prisons. The research team then worked with each healthcare team to identify staff from SPS, social work, external care companies and those that came in on a sessional basis such as GPs, forensic psychiatrists and psychologists. This process was used to get a sample of maximum variation that included staff from different disciplines; with different roles, insights, and perspectives to build up a situated and detailed picture of the current ways that people with a suspected dementia were identified, assessed and provided with post diagnostic care. We interviewed twenty staff.

Pragmatic sampling was used to reach men with diagnosed or suspected dementia and construct the case studies. The prison health care managers identified thirteen men with suspected or diagnosed dementia from health care records across the four prisons. The research team did not carry out any cognitive testing or assessment as all but one man with suspected dementia had undergone cognitive screening and all were undergoing further assessment. Five of the thirteen men did not meet the inclusion criteria due to being deemed to lack capacity or being too ill to participate. Eight men who were deemed to have capacity by the healthcare staff were approached, two declined and six gave their consent to meet with researchers. However, one man died before an interview was arranged. Five men were interviewed. The men were asked to nominate a significant other that could provide an insight into their lives. One man didn’t nominate anyone, however with his permission we interviewed his personal officer, four men nominated staff, and two men nominated the same member of staff. We interviewed four staff. All were asked and gave permission for prison health care staff to extract information from their health care records onto a questionnaire and provide it to the research team.

Convenience sampling was used for the workshops. Over a hundred stakeholders who the research team had communicated with before and during the study were invited to participate. Our Research Advisory Group also reached out to organisations and staff from criminal justice, social care and third sector community agencies who interface with those working and living in prison. Thirty eight individuals volunteered to participate. Participants included staff from a variety of disciplines and grades from the Scottish Prison Service and NHS, as well as staff from criminal justice, social care and third sector community agencies who interface with those working and living in prison, and people with lived experience of the justice system and dementia.

### Consent

All potential participants were provided with Participant Information sheets. They were reassured verbally and in writing that they had complete autonomy to participate or not, they could withdraw from the research at any time, participation or non-participation would have no bearing on their work role or relationships. After they indicated their understanding written consent was sought.

### Data collection

The twenty staff participants were given the choice to be interviewed online (*n* = 14) or face to face (*n* = 6). Interviews explored the current ways people with suspected dementia were identified and diagnosed, how their health and social care needs were met, what dementia education staff had and felt they needed.

The case study interviews were conducted face to face either in interview rooms within the healthcare centre or the unit where the prisoner was housed. Prisoners were escorted to the interview rooms by prison staff. To mitigate any risk posed to the researcher and to maintain confidentiality, a member of SPS or Healthcare staff was located, out of hearing distance but in the sight line of both parties. Interviews with men who had a diagnosed or suspected dementia explored what they recollected about how their cognitive issues were identified, how they were managing with activities of daily living, the support they received and thoughts about leaving prison. The interviews with the four case study staff explored their understanding of how the person was identified, assessed, their everyday life, enablers and barriers to providing care, interventions in place to promote their well-being, issues relating to risk, progression and release, and what dementia education they had and felt they needed.

The questionnaire data were extracted from healthcare records by nurses from the healthcare teams. It included a small biographical section, a section for past and current medications, and 25 questions about medical issues / conditions. Three data sources were combined to develop five case studies to provide an in-depth picture of the lived care experience of those with suspected or diagnosed dementia in prison. Interview and questionnaire data were gathered between June 2021 and November 2022.

### Analysis

We undertook a six phase Thematic Analysis (familiarisation, coding, generating initial themes, reviewing themes, defining and naming themes, writing up) to identify patterns of meaning within and across the interview data (Braun & Clarke, 2006). NVivo software was used; two members of the study team coded the data, reviewed, refined, and named themes to promote rigour by comparing levels of agreement. In addition, a cross case analysis with the data gathered from the men who had a diagnosed or suspected dementia was carried out to examine differences and similarities.

### Co-production

Following data collection we held three stakeholder workshops at which the high level findings and case studies (collated to illustrate typical experiences) were presented and checked for resonance with participants. The workshops were held in a central accessible venue designed for group events. Electronic feedback was captured, and notes of discussions were taken. We used an adapted World Café Method™ (2024) to facilitate in-depth focussed conversations. The prioritised actions were used to construct draft versions of the model and pathway which were sent out to the thirty eight workshop participants and the Research Advisory Group for review and comment (see Table 4).

## Findings

In this paper we are presenting an overview of the general findings from the interviews (*n* = 20) and the five case studies (see Tables 1, 2 and 3). We will also present the co-produced model and care pathway. The findings are presented thematically. Theme one the process, discusses the identification, assessment, diagnosis, care and support. Theme two the setting, discusses the environment and resources. Theme three the practice, which discusses the staff roles and responsibilities, communication, and training. Theme four the person, discusses the lived experience of having complex health and social care needs in prison and how the staff endeavoured to meet those needs.

**Table 2:** Number of interviews in each phase per prison

Number of interviews	Prison 1	Prison 2	Prison 3	Prison 4
Phase 1 staff	6	2	6	6
Phase 2 staff	2	0	1	1

**Table 3:** The profession of the staff interviewed in phase 1

Staff Participants	Phase 1
Primary Care nurses	3
Mental Health nurses	3
Prison social workers	2
Sessional GPs	4
Sessional Psychologists	1
Sessional Forensic Psychiatrists	1
Other Healthcare staff	2
Scottish Prison service staff	2
External carers	2

## The process: identification, assessment, diagnosis, care and support

### Identification, assessment, and diagnosis

There was no standardised way of identifying people with a suspected dementia within and across the four prisons. The national screening programme for prisons in Scotland does not currently include cognitive screening for older prisoners (HMIPs, n.d). There was no routine screening taking place in the four prisons in this study. Our findings suggested that healthcare staff were more reliant on those living in or working more closely with prisoners such as prison officers, prisoners, external carers or staff such as social workers alerting them to concerns.

*“I suppose we would rely a lot, unless we’re seeing people regularly, whether it’s for medications or if they have other medical issues, we would maybe pick up on things then, but other than that, you sort of rely on maybe other prisoners reporting things to the SPS, or the SPS picking up on these things themselves”.* Prison 3: Primary Care Nurse 2.

Once brought to the attention of healthcare, mental health nurses carried out an initial cognitive screen and history taking in consultation with a sessional forensic psychiatrist or GP. Staff reported that assessment processes were often complex due to several intersecting issues. There was a lack of clarity around the assessment processes, the nature of and resources afforded to providing healthcare in a prison setting, the poor health of this population and staff feeling they did not have the necessary expertise. We found none of the four prisons in this study had a care pathway in place at the start of the study and they all took a ‘case by case approach’. However, by the end of the study one prison had newly established an assessment and referral pathway in place with their local old age psychiatry team. Resource issues such as workforce shortages, particularly amongst mental health nurses and sessional staff could mean lengthy and disjointed assessment processes. COVID 19 had also placed additional pressures on staff with higher absence rates and additional restrictions adding to the time it took to carry out essential duties. All teams tried to take a collaborative multi-disciplinary approach to assessment referring to other health professionals to get a full picture of the person’s health and care needs. However, there was no lead for older adults either in SPS or prison healthcare. The need for prison healthcare staff to refer to community teams for input or assessment was common as specialist services such as occupational therapy (for people aged over 65 years), physiotherapy, speech and language therapy, audiology and old age psychiatry were not available within the prisons that participated in this study. Prison healthcare staff reported community teams responded to requests, albeit the wait times had lengthened because of COVID 19 restrictions. COVID 19 had also affected the ability of sessional support staff to come into the prison healthcare centres. The point in the assessment process that the four prison healthcare teams referred to old age psychiatry varied as did the support they received from old age psychiatry services in different Health boards. For example, one community old age psychiatry service had declined a referral submitted by the prison healthcare team on the grounds that they did not provide a service to people in prison.

*“I’ve gone through cycles of challenging the referral process. I did a standard referral through Old Age Psychiatry in the same manner I did only a few months earlier in the community for a similar gentleman. My community gentleman ended up being assessed, whereas my patient in prison within twenty-four hours, an e-mail following a telephone call to the health centre, came through: “We just don’t see patients in prison.” These should be referred to the forensic psychiatrists. In a prison health care setting, a lot of our patients have mental health issues and don’t have access to psychiatry as much as they should. People with a specialty should be working with a client and diagnosing it. Just as if somebody needed a cardiologist, they need a cardiologist. So that was disappointing though very unsurprising to me. But that case was a classic example of a referral that didn’t deviate in one way from a similar referral in the community with two separate outcomes”* Prison 1: GP 2.

This variation perhaps illustrates the national variation in practice amongst memory clinics, which provide clinical assessment for individuals with cognitive issues or suspected neurodegenerative disease (SIGN 168, 2023). Although the views varied as to when old age psychiatry should become involved in the assessment process, there was widespread agreement amongst prison healthcare teams that specialist input was required to support differential diagnosis. All the staff spoke of the challenges in identifying dementia or cognitive impairment: they didn’t know how an individual would ‘normally’ present, they had no baseline and felt they lacked the expertise to recognise signs and symptoms.

*“Because we don’t see these guys every day we don’t have baselines for these guys, we don’t know what their usual cognitive abilities are. Because obviously quite a lot of guys come into us do have head injuries and stuff. And then quite a lot of guys obviously have addiction issues as well, so it’s kind of a hard one when you’re trying to determine is it a cognitive impairment, is there something physical going on? Is there alcohol and drugs? So it is quite a hard one to kind of differentiate”.* Prison 1: Mental Health Nurse.

The ‘old’ age thresholds between the NHS and prison service differ. The Scottish Prison service tend to use the threshold of 50 or 55 years, whereas the NHS and local Authorities commonly use 65 years to define old age and services for older people. Although some dementia services will accept referrals of younger people with cognitive impairment this is not uniform (SIGN 168, 2023). This creates a challenge when it comes to those in prison accessing specialist services. The process for referring those with a suspected cognitive impairment who were under 65 years was unclear in all four prison healthcare teams.

### Care and support

To try and better meet health and social care needs of older men, the prisons in this study tried to accommodate older men and those with health and social care needs together. They also tried to allocate prison officers with more experience and or interest in working with this population to those areas. The extent to which prison officers became involved in providing social care and support varied between prisons and prison officers. This seemed related to whether there were external carers, a prisoner carer system in place and or the prison officers themselves. Personal care was provided by external carers contracted by SPS. Our findings suggest the external carers focussed on meeting personal care needs such as bathing, using the toilet and getting dressed rather than providing social care in its wider sense which would include support to stay active and participate in activities (Scottish Government 2021). The nursing staff wrote the care plans, and the external carers implemented them. The external carers could only provide the personal care specified in the care plan. If a person’s care needs escalated and the care plan was not updated to reflect increased care needs, those needs would go unmet until the care plan was updated. A shortage or high turnover of staff could result in care plans not being updated.

*“We've got one gentleman, whose dementia is at quite an advanced stage. If you ask him to do things he usually does the opposite. So we take our time, he needs help with everything, getting his food, dressing, getting showered. He needs a hoist now. The hoist needed its yearly maintenance check, it’s the only one we have and we asked and asked, it took weeks to get it done. He’s had an infection in his legs, I think, he was taken to hospital for tests, cos he couldn’t walk, and he came back unable to walk. He still doesn’t walk, he doesn’t stand. I have to put his food down in front of him and encourage him, once he gets started he can feed himself but he needs prompting”.* Prison 4: Carer 2.

On leaving prison the difficulties those with limited mobility, mental illness, and a disability are likely to have accessing supports such as housing, benefits and a GP is likely to be exacerbated (Scottish Government, 2021). For people with dementia, assessing their risk to the public when they were due for release was fraught with difficulty and complexity.

*“It (release planning) starts to become very complicated from our point of view, because we’re looking up what’s the nature of our client? What’s he actually in prison for? How are we also responding to risk? So it’s not just as simple forward saying, ‘oh gosh, he’s got dementia.’ We’re still having to think about what’s this individual’s response? How do we protect the public? the risk of re-offending. We’re having to worry about welfare needs and risk”.* Prison 1: Social Worker.

The ethical issues surrounding keeping people in prison when they no longer understand why they are there have long been of concern (Fazel, McMillan & O’Donnell, 2002). The quote below illustrates the challenges of supporting a person with advanced dementia in a prison setting.

*“The more it progresses, the measures aren’t, the prison environment isn’t conducive, it’s dangerous sometimes. We should be mirroring what’s happening in the community. And I totally appreciate that it is prisoners. However, everybody should be getting treated the same. And when you walk onto the sections up there, it is a prison. I mean, it must be so confusing, and you can see how scared some of them are. And you feel for the staff up on the halls because they don’t have the training, and they are trying their best, the hall staff up there are fab. But it’s difficult. One of them in particular just now, he’s just so confused and so agitated, and locked up all the time… and there’s no understanding about… if he is pacing, if he is in… I don’t know, it’s a difficult one, it’s just… we need to be mirroring what they’re doing in nursing homes, with the doors and the signage and making the environment more homely, and less distressing”.* Prison 3: Primary Care Nurse.

When an individual’s dementia advanced to point that they had complex, round the clock healthcare needs and were routinely becoming distressed, the prison healthcare teams would begin to look for alternative accommodation, unless the individual was on an Order of Lifelong Restriction. Finding suitable accommodation that would accept people with advanced dementia with high care needs who had current or spent convictions and whose risk to the public was difficult to determine was a challenging and protracted process, there appeared to be very few options available.

### The setting: environment and resources

As the previous quote illustrated the restrictive regime and built environment could be disabling and stressful. Prisons have few accessible cells, though modifications to the environment can be made. We found common adaptations were hospital beds, grab rails, commodes, and high back chairs. One prison had implemented several enabling features such as coloured tape around cell door, non-slip coloured mats for surfaces and contrasting coloured paint colour for two men who needed support with wayfinding, eating and mobilising. Organising modifications to the environment was often complex as it involved external specialist community staff and resource implications.

*“But again, it is – because of the limitations of the environment… so something that would be quite simple somewhere else, for instance arranging an appropriate diet… so the kitchen will take instruction from the nutritionist, who doesn’t come into the prison, who will only give advice, but doesn’t come in to assess people, and is reliant on our assessment. So there are assessments that we can do, but she will ask for a MUST [*an assessment to identify adults who are malnourished, at risk of malnutrition or obese*], which we are not all familiar with. And then the kitchen can respond with a modified diet. We do have a speech and language therapist who can assess swallowing and things like that. But it takes a prolonged period of time.*” Mental Health Nurse: Case study 4 and 5.

The prison healthcare staff were aware that psychosocial activities and supports to promote health and well-being available to many people living with dementia in the community were not available to those in prison. Often with poor mobility they were reported to spend most of their time on their own in their cells. None of the staff were aware of Scottish Government Local Delivery Plan standard on post diagnostic support (PDS) (Scottish Government, 2022a) or the PDS models being used to inform this work in the community (Scottish Government, 2017a).

Prison staff were there primarily to manage risk and safety. They tried to adapt regimes, if COVID restrictions and staffing allowed. People who became distressed when locked in their cell were sometimes permitted out of their cell to walk about during periods when others were locked up. However, there were a small number of reports that occasionally people with dementia, who were very agitated, and perceived as a threat to the health and safety of staff were restrained. This carer explains how having adequate staffing levels to provide care to someone experiencing high levels of agitation was a prerequisite to providing care safely.

*“To be honest with you, we don’t really get much quality time with that person because he poses too high a risk, he’s too unpredictable. He doesn’t make eye contact with anybody, his head’s down and he sort of grasps at his t-shirt as if it’s a kind of soother, and a comfort thing. And he’ll walk, and just keep walking, and he’ll do this for hours on end, every day. I don’t engage with him, it’s not because I don’t want to, it’s because I kind of don’t feel safe, we aren’t allowed to go into the section without a member of SPS staff. And again, you’re looking at staffing as well. They might not have the staff to allow for that. With a shortage of staff, prison staff, NHS staff, it’s quite… we’re just kind of.. I’m not saying winging it, but sometimes it feels you’re winging it, do you know what I mean? You’re trying to give really good patient care, but you struggle sometimes. I sometimes feel we’re quite ill equipped. And we’re trying to do the best we can, with the resources that we’ve got. But we really could be doing more”.* Prison 3: Carer 2.

### Practice: roles and responsibilities, communication, and training

The role staff should play in caring for people with dementia was sometimes disputed or unclear. Whose responsibility it was to provide a particular service and perceptions about the nursing role varied amongst SPS staff as this quote highlights.

*“Because some members of staff are quite resistant, saying ‘that’s not my job. You need to come and see him, he’s really confused today, you need to come and see him’. ‘No, if you use the reorientation stuff that I provided you’. ‘Well, where is it, I cannae find it. I don’t need to do this. This is your job, you do this.’ So depending on where you’re situated, depends on the buy-in from staff. So high healthcare needs for the sex offenders wing, the staff are there because they want to be there. Everywhere else in the jail is different. So it’s a bit complicated”*. Prison 3: Mental Health Nurse.

The notion of responsibility also appeared to be related to finance and resource. This was evident amongst services operating within and at the interface of the prison.

*“If they come from a different health board it is very difficult at times to get them to work alongside us. Because of all politics and funding”.* Prison 2: Mental Health Nurse.

Communication seemed impeded at times by the separate IT systems of the NHS and SPS. To enable information sharing between SPS and NHS people entering prison can be asked to give their ‘consent to share’. However, there seemed to be a lack of clarity about what information could be shared by whom under what circumstances. All the prison healthcare teams operated monthly multidisciplinary healthcare team meetings. Two of the prisons operated monthly interagency multidisciplinary healthcare meetings that involved prison staff for people ‘of concern’. In one prison it was reported that there was no interagency healthcare meeting due to a lack of buy in from the current senior prison management. All the staff felt interagency meetings supported communication and collaborative working between agencies. Some staff reflected on the reforms to prison healthcare and suggested these may have introduced tensions and divisions.

*“It’s very difficult as a nurse to be able to actually carry out your job to the best that you can because of the nature of the prison. I don’t know if every prison’s the same, but it just sort of seems to be becoming more of a divide. I think because it used to be like the nurses were all SPS, they were all officers as well and I think ever since the NHS has taken over that and obviously NHS has got its own protocols and processes and things that we have to do, I think that sort of gets a lot of people’s backs up. ‘Well, it’s a prison, you’ll kind of do what we say’. It’s a very difficult place to work (laugh).”* Prison 3: Primary Care Nurse 2.

All the prison healthcare staff reported challenges communicating with the many and various Health Boards, health and social partnerships and local authority services from across Scotland. Although prison healthcare staff generally knew the services and contacts within their local area, it was not possible to have this knowledge for services across Scotland. Most of the staff felt that having identified leads in each prison for older prisoners in SPS, NHS and the local Health and Social Care partnerships would support interagency care planning.

All the staff felt unequipped to work with people with dementia. None of the staff were aware of the National Education for Scotland (NES) Promoting Excellence dementia education resources freely available to all staff working in health and social care settings. None of the prison or prison healthcare staff reported having had any dementia education or training since taking up their current post. Although some of the prison healthcare staff had previously had some dementia education they didn’t feel they had the appropriate skills, knowledge and expertise in dementia to identify someone with cognitive impairment, discuss or provide a diagnosis of dementia or know what care and support would be helpful post diagnosis.

*“So I suppose we would kind of link in with community services to think about those cases where we might need either potentially in-reach or consultation, to get some support. Because although we are trained to assess people’s cognitive functioning, it sort of feels like there’s a point where you need that more expert involvement. I mean, to be honest, I think the biggest thing that needs to be worked on is the interface between kind of prison health care and community services. I think dementia would be an example of when we would be looking for elite or kind of specialist service involvement.”* Prison 1: Psychologist.

### The person: complex health and social care needs

The case studies illustrated the poor health so often attributed to the prison population generally and more specifically older prisoners. The age range of the men was 60–79 years, four were over 65 years. All were on long term sentences (over 4 years). All had multiple co-morbidities, this ranged from 3–8, all were prescribed multiple medications, this ranged from 4–11. Two of the men were in receipt of personal care provided by external carers. This meant they had been assessed by the nursing team as requiring support to meet their fundamental care needs such as washing, dressing etc. Three had significant mobility issues and either used a walking frame and or wheelchair to mobilise. Two were accommodated in accessible cells and one had an airbed. Four of the five men could not participate in work programmes, and needed assistance if they wanted to go outside or visit the library. They largely stayed in their cell or the immediate area, relied on other prisoners to remind them or help them with ordering, cleaning their cell, or occasionally going outside for exercise. For these four men their greatest concern was around their deteriorating physical health and for two of them it was about maintaining contact with family members.

*“I fall about, because I’m… I’ve no balance, and I keep telling them, I keep telling them and saying, “Look, I’ve no balance at all,” ken? “Oh right, right (name of person).” But nothing gets done. Honestly, and it’s so irritating. It’s difficult for me to move about myself, you know? That’s terrible. I’m using that, this walker here, mainly. Well I’m… well it’s quite difficult, especially in the cell because there’s obstacles, you know? And you have to hang onto the sinks and hang onto the back of chairs and everything. I mean, I’m just… I’m no’ doing well in my cell”.* Fraser: Case study 3.

Prison and prison healthcare staff had different philosophies of care, the most obvious illustration being the way people in prison were defined, they were patients to the prison healthcare teams and prisoners to the SPS staff. The prison healthcare staff while recognising health and safety saw these situations through the prism of unmet care needs whereas prison staff tended to view these situations through the prism of staff safety as this quote illustrates.

*“Where the unpredictability and the violence was there, we have to make sure that he’s protected. So we’re very kind of switched onto that, the staff are very experienced in managing that. I suppose another hindrance was, the prisoner’s behaviours. I think, albeit we have small pockets of guys with this kind of early stages of dementia and cognitive ability, but rightly or wrongly you treat a violent prisoner as a violent prisoner, you’ve got to kind of look at the health and safety of the staff as paramount.”* Prison 3: Senior Prison Officer.

The following quote picks up on issues discussed earlier such as roles and responsibilities and the lack of education and understanding. It highlights the importance of prison staff having enough understanding and skills to better manage escalating distress and raises the issue of how people can be better supported in this environment.

*“Because they’ve (SPS) probably seen that, as in years ago, ‘oh that’s not our job, that’s health care.’ But it’s going to have to be a certain type of prison officer that is going to deal with these patients. Because really they have a duty of care as well, as much as what we do, that you want to be able to have the right mix and, you know, people actually want to care, so to speak for them- for these patients. And sometimes I feel… not all members of SPS staff, but there is a lot of new starts that have just came in, that say for example the first gentleman, if he continues to bang on the door, because he sees it as being daylight, why is he being locked up? He doesn’t quite see it, he sees the door as being an obstacle, he needs to get out. You’ll have other members of staff that are just not aware, or know how to deal with him, and they’ll start shouting back at him, so you’re only escalating his behaviour even worse.”* Prison 3: Support Worker 2.

Escalating care needs was also a topic prison healthcare staff returned to often. Although those with advanced dementia were not included in case studies, staff talked about the small number of individuals who required very high levels of care 24 hours a day. These individuals no longer had capacity, spent most of their time in bed, required continence care, pressure area care, support to wash, dress, eat, drink and a hoist to move from bed to chair.

### Co-produced outputs: model and care pathway

The workshops provided an opportunity for participants to engage with the high-level findings and two composite case studies. Following the world café method™ the group articulated principles, actions and processes they felt were needed to create a model of care and a pre- and post-diagnostic care pathway. Table 4 illustrates the participant prioritised actions, processes and activities that would support the operationalisation of a pre- and post-diagnostic care pathway.

**Table 4:** Stakeholder workshop questions and participant prioritised actions, processes and activities

Question/issue	Action
How can we improve processes for identifying and assessing prisoners with suspected cognitive impairment or dementia?	Consider introduction of tailored cognitive screening for older people in prison Routinely get consent to share early to streamline the sharing of information Record cognitive impairment in both NHS and SPS information systems Training on GDPR and information sharing Dementia training and education for all staff Have an NHS, SPS and Local authority adult services or HSCP lead that has responsibility for older adults/people with cognitive impairment
How can we develop a diagnostic pathway that includes input from specialist community services?	Develop and implement a clear referral and care pathway with staff roles and responsibilities Improve access to and links with community services and specialists (such as old age psychiatry and older adult social work) Greater use of technology could enable more specialist services input into prison Increase understanding about criminal justice, adult and older adult social work role and responsibility Identify NHS, SPS and HSCP leads to support coordination, improve accountability and responsibility Develop more collaborative working with Third sector providers to better support staff, prisoners and families
How can we improve multidisciplinary care planning to address individual physical and mental and care needs?	Develop and implement a clear referral and care pathway with roles and responsibilities of SPS and NHS in place Responsible/named person to support integrated care management. Care programme approach (CPA) focuses on health which means less focus on risk. Integrated Case management (ICM) would focus on care and risk Identify NHS, SPS and HSCP leads to support coordination, improve accountability and responsibility
How can we improve multidisciplinary care planning to address individuals needs for reasonable adjustments, and participation in social and recreational activities?	Need access to staff with knowledge and skills to support social and recreational participation Need access to staff with knowledge and skills to identify required reasonable adjustments Training on GDPR, confidentiality and information sharing to support interagency working Improved interagency working and communication Government strategies need to recognise that there are people living in prison with dementia and they have health and social care needs Build design of new prisons needs to take account of ageing population. Money to retrofit and increase number of accessible cells
How can we improve multidisciplinary care planning to prepare and support individuals for progression and release?	Increase staff understanding the referral pathways within and at the interface of prison and the community Ensure all staff know their roles and responsibilities Improve interagency working, information sharing and communication Have identified multi agency leads with accountability and responsibility to support partnership working Prison social care costs increasing, need to support safe timely release

From the agreed actions of workshop participants the research team drafted a model and care pathway. The team also took account of the evidence from this study and the most comparable recent study conducted in England (Forsyth et al., 2020). It was also important to be cognisant of the HMIPs Inspecting and Monitoring: Standard 9: Health and Wellbeing and the National Health and Social Care Standards (Scottish Government, 2017b), both of which adopt a human rights based and person centred approach to care and treatment. Two subsequent successive drafts of the model and care pathway were reviewed and commented on by workshop participants and the Research Advisory Group.

**Fig. 1:**
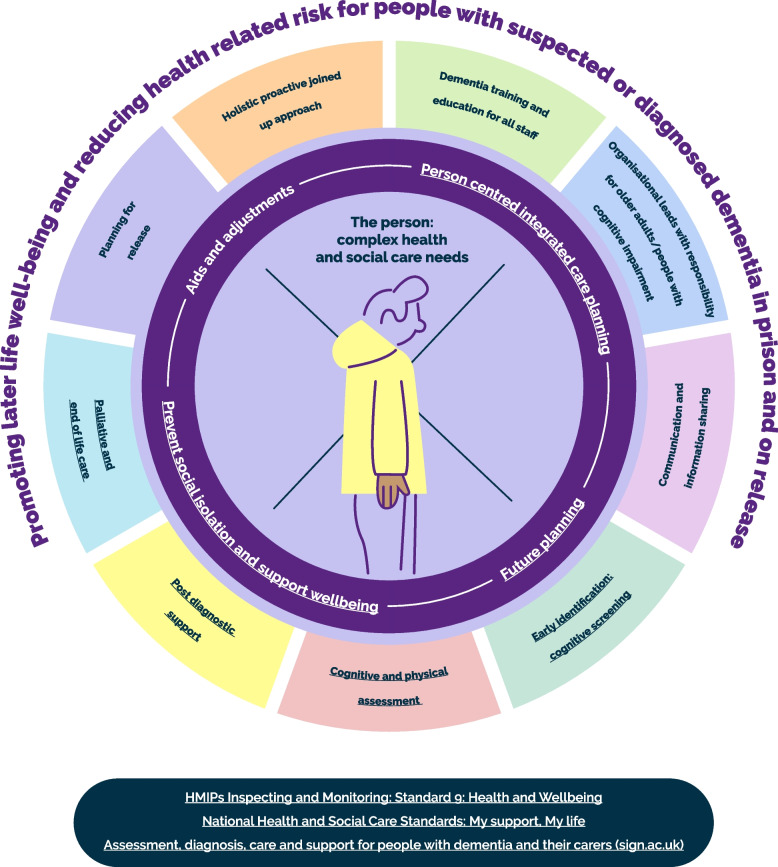
Care model for pre- and post-diagnostic care of people with suspected or diagnosed dementia in prison HMIPs Inspecting and Monitoring: Standard 9: Health and Wellbeing (https://prisonsinspectoratescotland.gov.uk/publications/inspecting-and-monitoring-standard-9-health-and-wellbeing) National Health and Social Care Standards: My support, My life (https://www.gov.scot/publications/health-social-care-standards-support-life/pages/2/) Assessment, diagnosis, care and support for people with dementia and their carers (sign.ac.uk) (https://www.sign.ac.uk/our-guidelines/assessment-diagnosis-care-and-support-for-people-with-dementia-and-their-carers/)

The overarching aim of the model below is to represent the care principles to ensure the well-being of people with suspected or diagnosed dementia in prison and on release (Fig. 1).

**Fig. 2:**
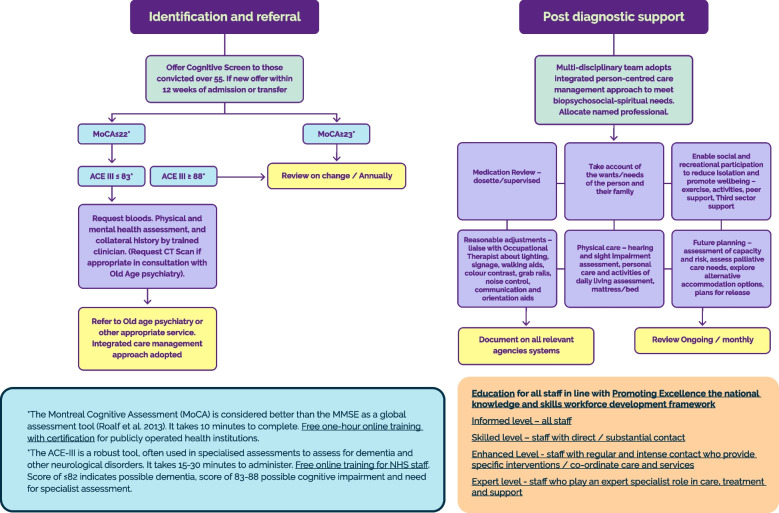
Care pathway to operationalise the identification, assessment, diagnosis, post diagnostic care and support for people with a suspected or diagnosed dementia in prison *The Montreal Cognitive Assessment (MoCA) is considered better than the MMSE as a global assessment tool (Roalf et al. 2013). It takes 10 minutes to complete. Free one-hour online training with certification (https://mocacognition.com/training-certification) for publicly operated health institutions *The ACE-III is a robust tool, often used in specialised assessments to assess for dementia and other neurological disorders. It takes 15-30 minutes to administer. Free online training for NHS staff (https://www.mvls.gla.ac.uk/aceiiitrainer). Score of ≤82 indicates possible dementia, score of 83-88 possible cognitive impairment and need for specialist assessment Education for all staff in line with Promoting Excellence the national knowledge and skills workforce development framework (https://www.gov.scot/binaries/content/documents/govscot/publications/advice-and-guidance/2021/05/promoting-excellence-2021-framework-health-social-services-staff-working-people-dementia-families-carers/documents/promoting-excellence-2021-framework-health-social-services-staff-working-people-dementia-families-carers/promoting-excellence-2021-framework-health-social-services-staff-working-people-dementia-families-carers/govscot%3Adocument/promoting-excellence-2021-framework-health-social-services-staff-working-people-dementia-families-carers.pdf) Informed level – all staff (https://www.bing.com/videos/search?q=informed+about+dementia+dvd&docid=603507248022495397&mid=7F9AA11BFAE6C52D494F7F9AA11BFAE6C52D494F&view=detail&FORM=VIRE) Skilled level – staff with direct / substantial contact (https://www.nes.scot.nhs.uk/media/lp1hh5kt/dementia-skilled-resource-2016-final-web.pdf) Enhanced Level - staff with regular and intense contact who provide specific interventions / co-ordinate care and services (https://www.nes.scot.nhs.uk/media/zw0o3utc/promoting-psychological-wellbeing-for-people-with-dementia.pdf) Expert level - staff who play an expert specialist role in care, treatment and support (https://www.gov.scot/binaries/content/documents/govscot/publications/advice-and-guidance/2021/05/promoting-excellence-2021-framework-health-social-services-staff-working-people-dementia-families-carers/documents/promoting-excellence-2021-framework-health-social-services-staff-working-people-dementia-families-carers/promoting-excellence-2021-framework-health-social-services-staff-working-people-dementia-families-carers/govscot%3Adocument/promoting-excellence-2021-framework-health-social-services-staff-working-people-dementia-families-carers.pdf)

The overarching aim of the evidence informed coproduced pathway is to provide staff with a resource to guide the identification, assessment, diagnosis, post diagnostic care and support for people with a suspected or diagnosed cognitive impairment or dementia (Fig. 2).

## Discussion

### Merits and limitations

This study included four of the 15 prisons in Scotland. The four prisons had 2,936 (39%) of the combined population of 7,504 in 2021/22 (Scottish Government, 2022b) and were selected on the basis that they housed the largest numbers of men over 65 years. This is justifiable as age is an important risk factor for dementia and it follows that dementia related needs are more likely to be concentrated within these four sites. All thirteen men identified in the four prisons were over 60 years, so our findings are limited in that respect. The research was conducted between June 2021 and November 2022, there was a six-month data collection pause due to the impact of COVID-19 on prison healthcare staffing levels. There are known variations across the prison estate in terms of the healthcare needs of each prison population, the profile and numbers in each prison healthcare team and their approach to identification, assessment, and diagnosis of people with suspected cognitive impairment or dementia. Although the sampling strategy may reduce generalisability of findings, it nonetheless provides evidence critical to understanding the adequacy of current approaches to meeting dementia related needs within a prison setting.

### Screening for cognitive impairment

The findings suggest there was no early or ongoing screening for cognitive impairment. Prison health care staff were largely reliant on other staff or prisoners, who had no education or training in identifying cognitive impairment, alerting them to concerns. This in effect meant that people were being identified by chance when someone that knew them well enough reported changes or when they became a management concern. This is a missed opportunity for early identification that would trigger an assessment process. For these reasons we would recommend in line with other authors the introduction of systematic cognitive screening programmes for older prisoners (Combalbert et al., 2018; Stolkier et al., 2022; Brooke et al., 2020; Peacock et al., 2020). Although time and resource are not insignificant challenges, cognitive screening happens in 30% of English prisons (Forsyth et al., 2020). Furthermore, recent work found that professionals working in Scottish prisons felt that it would be feasible to screen the existing over 55 years population and conduct cognitive screening in sentenced prisoners over 55 years within 12 weeks of admission (MacRae, 2024).

We would also recommend along with others that cognitive screening tools be validated for use in prisons (du Toit., 2019; Forsyth et al., 2020; Ahalt et al., 2018). Diagnosis of dementia in this population is complex due to the high prevalence of head injury (McGinley et al., 2019; Worthington et al., 2017), presence of other co-morbidities (Hayes et al., 2012, 2013) and low educational attainment (Ahalt et al., 2018). The prison health care staff felt they should not stretch beyond their educational and disciplinary competencies to undertake specialist diagnostic assessments. We would argue that input from specialist community services such as old age psychiatry are much needed for people, both under and over 65 years, who have been identified with cognitive impairment in prison to be given a timely and differential diagnosis.

### Assessment and care planning

We found that multidisciplinary assessment and care planning processes were in place. However, they did not always include prison staff. Previous research has shown the benefit of early need assessments to achieve better outcomes (Forsyth et al., 2020), the important role social workers can play in care planning (Ruggiano et al., 2016) and the benefits of an integrated team model for palliative care (Maschi et al., 2014). We would recommend that following diagnosis, a formal assessment of a person’s biopsychosocial-spiritual needs by a health, allied health or social care professional with the appropriate dementia knowledge and skills should be carried out. This would support the creation of a package of care, we would advocate for this to be inclusive of the person and their family. Any package of care needs to support social and recreational participation and reasonable adjustments if wellbeing is to be improved. Our findings would support previous recommendations that advocates for equal access to participate in leisure activities, education and programmes that support progression (Brooke et al., 2020; Dillon et al., 2018) which will in turn support the assessment of risk. Having staff available that can assess, facilitate and implement these supports is important if we want to prevent symptoms from being misinterpreted as symptomatic of other mental health issues or disobedience, minimise distress, improve the consistency and quality of physical, emotional, and social care support in prison and on release (Di Lorito et al., 2018; Williams et al., 2012).

### Leadership and collaboration

There were no designated leads for older prisoners in SPS or NHS healthcare teams. In contrast over half (59%) prison healthcare teams in England reported having an identified older prisoner lead (Forsyth et al., 2020). Many staff felt that due to the age and disability of people with cognitive impairment or dementia, they could fall under the equality and diversity remit in prisons. All the staff felt there should be designated NHS, SPS and Social work or Health and Social Care Partnership agency leads who could support multi agency working within and at the interface of prison. It was felt that strong interagency leadership was one way to support a multi-disciplinary approach and interagency collaboration to drive forward improvements in pre and post diagnostic care and staff education. Specifically the streamlining of intra and inter agency information sharing, clarity about intra and inter agency processes and responsibilities, the establishment and or maintenance of interagency healthcare meetings to support communication, care planning, care in custody, and early consideration of risk, care and social participation needs to support transfer or release. If established these may go some way to managing the tensions between very differing philosophies of care and security.

### Complex and palliative care

Dementia is a neuroprogressive illness, thus it has been argued elsewhere that dementia specific palliative approaches to care are necessary (Lewis et al., 2023). A recent scoping review of dementia care pathways in prison suggested that a spectrum of healthcare including long term and palliative care should be delivered (Treacy et al., 2024). It also revealed a long-standing debate about the detrimental or beneficial effects of separate and or specialist or integrated accommodation for people with dementia in prison (Treacy et al., 2024). Our findings suggested that the prison healthcare staff felt that it was inappropriate to keep people with advanced dementia in prison. Palliative dementia care requires a range of specialists with the prerequisite knowledge and skills to provide the necessary care for the many distressing symptoms such as pain, sleep disturbances, problems with eating and swallowing, agitation and infections that come with advanced dementia (Eisenmann et al., 2020). Knowing the complex care needs of these individuals were not being met the healthcare staff tried to find alternative accommodation in the community, however this proved very challenging and supports previous research that this can delay or prevent release (The Correctional Investigator Canada, 2019). This begs the question, where can people living with advanced dementia, who lack capacity, who may or may not pose a risk to the public be accommodated and provided with palliative dementia care? We suggest further consideration needs to be given to secure nursing provision.

### Workforce Education

Our findings suggest there is inadequate knowledge amongst prison and prison healthcare staff about cognitive impairment, dementia and ageing. This lack left the staff feeling unequipped and lacking in confidence to support older people and people with diagnosed or suspected dementia. Our findings suggest that this hampered the identification, assessment, diagnosis and post diagnostic care. We recommend that staff are supported to engage with the freely available dementia education that may go some way to equipping them with the knowledge and skills to respond appropriately to those with suspected or diagnosed dementia. Literature reviews on caring for older populations in prison are replete with recommendations that staff need more access to education so they can feel competent and provide appropriate care (du Toit et al., 2019; Hagos et al., 2022). Our findings suggest that staff also need more information and support from their employing organisations. They wanted more clarity about inter agency information sharing and referral processes, understanding of one another’s roles, responsibilities and multi-agency working.

### Bridging the gap: policy and practice silos

Our findings are set within the context outlined in previous reports which found prison health care understaffed and under resourced (House of Commons Health & Social Care Committee, 2018; Ismail, 2020; Mental Welfare Commission, 2022). Many of our findings chime with previous reports that have outlined the strategic and operational issues with resource allocation to prison health care and associated challenges in meeting the complex physical and mental health care needs of the populations they serve (HMIPS, 2017; Gaston, 2018; Mental Welfare Commission, 2022; Gilling-McIntosh, 2023). The sustained increase in older prisoners calls for a whole system approach to meeting complex health and social care needs of older prisoners, a substantial number of whom will have cognitive impairment or dementia. Current government policies and strategies are either for dementia or prison, neither take account of people living in prison with a diagnosed or suspected dementia. If we continue to have siloed policy and practice we risk the continuation of care that is inconsistent, institutionally thoughtless and neglectful (Crawley, 2005; Prisons and Probation Ombudsman, 2016; Williams, 2012; HM Inspectorate of Prisons and Care Quality Commission, 2018). In the absence of policy or financial support from governments we are reliant on leadership teams in prison, prison healthcare and local health and social care teams to work together to adapt and improve existing approaches to care in custody, engage with dementia education and the recommendations coming from research.

## Conclusion

Our study adds to what is known about how people with suspected cognitive impairment or dementia are identified, assessed, diagnosed and provided with post diagnostic care in prisons. It makes policy and practice suggestions about how to improve care for this marginalised group. Importantly it offers a model of care and pre and post diagnostic care pathway that was co-produced with stakeholders working in or at the interface of prisons and prison healthcare. The model and pathway offer an operationally practical flexible resource to inform and guide an integrated person centred approach to pre- and post-diagnostic dementia care in prison. If implemented the pathway and model could address many of the hitherto cited problems and gaps in care such as processes for identifying cognitive impairment and onward referral, and a model for multidisciplinary patient centred care and support for this vulnerable population. We would suggest that next steps include working with prisons and prison healthcare teams to implement the pathway to test and refine it in context.

## Data Availability

The datasets used and or analysed during the current study are available from the corresponding author on reasonable request.
